# First things first: An exploration of the effects of psychoeducation for older autistic adults

**DOI:** 10.1177/13623613231219745

**Published:** 2024-01-10

**Authors:** Maartje Lenders, Machteld A Ouwens, Rosalien M H J Wilting, Arjan C Videler

**Affiliations:** 1 GGz Breburg, The Netherlands; 2 Tilburg University, The Netherlands

**Keywords:** autism spectrum disorders, interventions—psychosocial/behavioral, older adults, psychoeducation

## Abstract

A psychoeducation program that was originally developed by the Dutch Association for Autism was adjusted for older adults to enhance its feasibility and efficacy in later life. In this study, we explored the effects of this adapted psychoeducation program for older autistic adults in a pre-test–post-test 6-month follow-up design. A total of 61 patients (55–78 years) and 44 proxies (a person close to them) participated in the study; attrition rate was high for the follow-up assessment. As hypothesized, an increase in knowledge and acceptance of the diagnosis was observed, with a strong and positive correlation between patients and proxies for knowledge. Furthermore, ambiguous results were observed on coping with the diagnosis and no positive effects on psychological distress were found. The feedback of participants and proxies about the psychoeducation program was largely positive. Future research with larger group samples and larger time scales is necessary to gain more insight into the effectiveness of the psychoeducation program. It seems worthwhile to further adjust the program to the needs and requirements of older adults, and to help older autistic adults to construct a new narrative of themselves, and the life they have lived, in the light of the recent autism diagnosis.

**Lay abstract:**

After receiving an autism diagnosis, psychoeducation (i.e. information regarding autism) is a first intervention. We adjusted a psychoeducation program that was originally developed by the Dutch Association for Autism for older adults to enhance its feasibility and efficacy in later life. We expected that participants would report an increase in knowledge and acceptance of the diagnosis and that people close to them would also observe this. Indeed, we found this and participants and those close to them agreed on this. Furthermore, we found some evidence that older autistic adults were better at coping with their autism. We found no positive intervention effects on psychological distress. The feedback of participants and informants about the psychoeducation program was largely positive. In future research, we advise using larger group samples and larger time scales and we also advise to further adjust the program to the needs and requirements of older adults, and to help older autistic adults to construct a new narrative of themselves, and the life they have lived, in the light of the recent autism diagnosis.

## Introduction

Autism has been severely underdetected in older adults until recently (Robison, 2019). Because of growing attention for autism in later life, detection and diagnosis has increased in older adults (Patra, 2016; Robison, 2019). National guidelines for autism, such as the Dutch Multidisciplinary Guideline Autism (Kan et al., 2013) and the British NICE guideline (2021), advise to start with psychoeducation (PE) as a first step in treatment, directly after someone has been diagnosed. They also advise to involve family members, friends, or loved ones in the PE program. Despite these recommendations, access to PE for older adults with a recent diagnosis of autism is severely hampered in the Netherlands: to the best of our knowledge, only six specialized mental healthcare institutes provide PE for older autistic adults across the country; also internationally, PE for older autistic adults seems to be scarce.

PE is an important first intervention, before specific personalized interventions start. PE is a systematically deployed and structured form of patient information and education, with the aim of informing patients and their family members about the etiology of a condition or illness, triggering factors, coping with the condition and options for treatment (Kumar et al., 2015), which helps people to make the best health-promoting decisions (Pekkala & Merinder, 2002). Furthermore in a group program there is the advantage of contact with fellow patients, which gives a feeling of not being alone, support, and understanding. There is evidence for the efficacy of PE for patients with several psychiatric disorders and somatic illnesses, and some evidence for the efficacy of PE for autistic people. PE interventions help reduce symptomatology and increase treatment outcomes for patients with physical illnesses, for example, cancer (Cunningham, 2000; Lukens & McFarlane, 2004). For patients suffering from psychosis/schizophrenia, PE has been associated with lower admittance and rehospitalization rates (Bauml et al., 2006), with improved compliance and reduced relapse (Sin et al., 2017), and improved quality of life (Xia et al., 2011). For patients with bipolar disorder, PE was found to be efficacious in the prevention of all types of relapse (Camardese et al., 2018).

Although PE is recommended as first intervention after people receive an autism diagnosis, studies into the effects are relatively scarce. Vermeulen (2000) has developed a PE program “I am special,” widely used in the Netherlands and Belgium for younger adults in several adaptations, with little data on its efficacy. A study by Kan and colleagues (2008) in eight consecutive PE groups of six autistic adults and their partners found that participating in a 13-week PE program resulted in a significant increase in knowledge about autism for both autistic adults and their relatives, and a significant increase in recognizing autistic traits among the autistic adults. An online survey by Spek and Boxhoorn (2014) with 130, mostly female, autistic participants between 18 and 60 years old found that PE was perceived helpful in accepting the diagnosis by 19% of the female participants and 24% of the male participants. Reading about autism and contact with other autistic adults was experienced as most helpful in accepting the diagnosis, suggesting there is room for improvement in how PE is delivered. However, it was not reported by whom and how PE was delivered to the participants. A randomized controlled trial by Gordon et al. (2015) detected a significant effect of a 6-week PE intervention on knowledge of autism and on autism-related self-awareness in young autistic individuals. In autistic adolescents, knowledge and self-awareness regarding autism-related strengths and difficulties increased in most participants from pre- to post-treatment after following the PEGASUS-PE program; however, most results were not significant (Gordon et al., 2015). A study by Smith DaWalt and colleagues (2021) into a group PE intervention “Working together for adults with autism spectrum disorder” found significant increases in meaningful activities and decreases in internalizing problems after following the 6-month multifamily group intervention.

A Delphi study concerning assessment and treatment of autism in older adults (Hitzert et al., 2016) yielded consensus among experts in autism in geriatric psychiatry that the standard PE program for adults with autism should be adjusted for older adults; for example, it should take into account sensory disabilities, changes in information processing, and reduced motor skills in older adults in comparison with younger cohorts. Also, it was considered necessary to integrate other topics that are relevant for older adults, for example, physical health and practical support at home (Heijnen-Kohl et al., 2018; Spek & Geven, 2013).

There are few empirical data on the effectiveness of PE for older adults with a late autism diagnosis. A qualitative interview study by Boland and colleagues (2017) found that the majority of eight older autistic participants that received PE-reported positive changes, such as a reconfiguration of their self-image and a better appreciation of their individual needs. Groenendijk and colleagues (2022) explored the effects of a co-designed PE program for older autistic adults (55+) in a multiple case study (*N* = 9). For each participant, a proxy participated as well. The results of this study showed no significant improvement regarding insight in autistic traits nor in cognitive challenges, nor on the secondary outcome measures (mastery, self-esteem, quality of life, self-efficacy, self-stigmatization and hope, and future perspectives). However, the authors stated that, based on the positive feedback and suggestions of the participants in this study, developing an improved version of a specific PE program for older adults with autism is a worthwhile pursuit. The PE program in this multiple case study, however, followed the regular PE so it is probably not directly comparable to effects obtained with a PE which directly follows a recent diagnosis. An exploratory study into a brief PE group intervention for partners of older adults with a recent autism diagnosis, “Living together at your own strength,” found that partners reported increases in knowledge about autism, recognition of autism in their partner, and in self-esteem, and a decrease of psychological distress (Ouwens et al., 2022).

### Aim of the current study

In sum, little is known about the efficacy of PE for autistic older adults. Because detection and diagnosis of autism in older adults is improving, there is an increasing need for adequate and helpful PE for older adults with a late autism diagnosis. This study was a first exploration of the effects of an adjusted PE program for older adults. The main aim of this study was to explore the feasibility and effects of the Dutch PE program “Me and Autism,” adapted for older adults on knowledge about, acceptance, and understanding of the diagnosis, coping with autism and comorbid general psychological distress, as perceived by participants and people close to them (proxies).

The concordance between informant-report and self-report for assessing autistic adults has been found to be moderate (Sandercock et al., 2020; Taylor et al., 2022). There are several reasons why self- and informant-reporting may differ, including some aspects of autism being internal states and not directly observable, observer bias (e.g. Obeid et al., 2021), and camouflaging (Cook et al., 2021). Both informants and autistic adults provide valuable but different information when reporting on symptoms and experiences. Therefore, the second aim of this study was to examine the difference in how patients and their proxies (mostly relatives) experience the effect of the program.

## Methods

### Trial design

A pre-test–post-test follow-up design was used for participants and proxies. Measurements were taken at the start of the program and at the last session, and follow-up data were gathered 6 months later. The study was approved by the ethics committee of GGz Breburg.

### Participants and recruitment

The program “Me and Autism 60+” is part of treatment as usual for older adults diagnosed with autism at PersonaCura, clinical center of expertise for personality disorders and autism in older adults of GGz Breburg, and at SeneVita GGz. Participants were recruited among the participants between May 2018 and May 2021. Participants were older adults, recently (<6 months) diagnosed with autism by a multidisciplinary team according to the Dutch Multidisciplinary Guideline, as confirmed by the *Diagnostic and Statistical Manual of Mental Disorders* (5th ed., *DSM*-5) interview for autism (Spek, n.d.). Informants (proxies) were approached through the participants and filled in the questionnaires at the same measuring points. The PE program “Me and Autism 60+” was developed for older adults who receive a late autism diagnosis at the age of 60 years and older; however, participants were included from the age of 55 and above. Exclusion criteria were a comorbid psychiatric disorder that needed acute treatment (e.g. psychosis); a neurocognitive disorder (e.g. dementia); substance use disorder, which required detoxification; and/or an IQ below 70. These exclusion criteria were assessed after extensive psychiatric and psychological assessment by the multidisciplinary teams.

### Procedure

Participants were recruited during the first PE session. One of the researchers joined at the end of the first session and gave information about the study. After informed consent was obtained, participants filled in the questionnaires. Measures were delivered as a paper and pencil version and distributed by the research team at the first and second measurement. After the session, participants invited a proxy of their choice to also participate in the study after their informed consent was obtained. At the penultimate session, participants received the questionnaires for the second measurement. Participants themselves gave the questionnaires for the second measurement to the proxy of their choice. The 6-month follow-up questionnaires for participants and proxies were sent by post to the participants with the request to distribute the informant questionnaires to their proxy, and to return them by post.

### Intervention

#### “Me and Autism 60+” PE program

The program “Me and Autism 60+” consists of seven weekly group meetings of 2.5 h each, an eighth session in which a person close to them of choice was invited, and one reunion session after 1 month. Maximum participants per group was eight (during the Covid-19 pandemic, this was temporarily reduced to six because of social distancing measures; furthermore, it was temporarily not possible for participants to invite a proxy to the eighth session of the program). Topics are knowledge (about autism, consequences of autism, treatment options, and social learning) and acceptance of the diagnosis. See Table 1 for a more detailed overview of the themes covered by the program. The adjustments for older adults were adjustments in the content of the program, considering age-related cognitive decline, and more time for peer support.

**Table 1.** Content of the weekly sessions of the psychoeducational program “Me and Autism 60+.”

Week	Theme	Content
1	Observation and stimulus processing	Acquaintance with group members and trainers, differences in observation, and stimulus processing
2	Information processing	Differences in information processing
3	Emotions	Elements of cognitive therapy, basic emotions
4	Communication	Effect of autism on communication
5	Diagnosis	Theory of mind. central coherence, executive functioning
6	Daily life	Health, living, daytime activities, social network, compensation techniques
7	Integration and evaluation	
8	Informants	Theories about autism, do’s and don’ts

Participants received a workbook containing information and assignments. In between meetings, participants had to read texts and complete assignments, as preparation for the next session. Trainers were experienced specialized nurses or social workers. In order to determine treatment adherence, trainers were given a checklist, in which they indicated each session which part of the manual was discussed.

### Outcome measures

#### Self-report measures

##### Primary outcome measures

*A self-developed 36-item questionnaire* was used to assess acceptance and knowledge of the diagnosis, and coping with autistic traits. Qualitative open questions were added to investigate the participants’ and their informants’ opinions about the PE program in more detail. This quantitative and qualitative measure was developed by the authors in co-design with autistic older adults who were also peer supporters. There are no data available yet about the reliability and the validity of this questionnaire.

Knowledge about autism was measured by 21 items, for example, “I have knowledge about autism” and “I know when my behavior is consistent with autism.” Patients scored the questions on a 5-point scale (“totally not true,” “more not true than true,” “more true than not true,” “totally true,” and “I don’t know the answer”). There are also two open questions that measure knowledge of autism, for example, “What more have you learned during the program?”

Acceptance of the autism diagnosis was measured by two items. The item “I can live with my autism diagnosis” is scored on a 5-point scale (“totally not true,” “more not true than true,” “more true than not true,” “totally true,” and “I don’t know the answer”). The item “I accept my autism diagnosis” is scored by “yes” or “no.”

Coping with autistic traits was assessed by 11 items. Participants had to determine how often they recognized several events, for example, “I recognize my autistic behavior in everyday life” and “I’m prepared to listen to the advice I’m receiving about how to cope with problems.” These items are scored on a 5-point scale, ranging from “never” to “very often.”

The Dutch version of the Social Responsiveness Scale-Adults (SRS-A) (Constantino & Gruber, 2016) was used to assess autistic traits. This questionnaire consists of 64 items, scored on a 4-point scale, ranging from “not true” to “mostly true,” directed at the different dimensions of interpersonal behavior, communication and repetitive/stereotypical behavior, characteristic for autism. The questionnaire consists of four subscales: Social Awareness (SA), Social Communication (SC), Social Motivation (SM), and Repetitiveness and Rigidity (RR). The questionnaire is developed for adults, aged 18 to 65, and is used mainly for screening, but it can also measure change in autistic traits. The Dutch version is highly reliable (internal consistency: Cronbach’s alpha = 0.96, subscales between 0.79 and 0.88; test–retest reliability: r = 0.88, for subscales between 0.70 and 0.86).

##### Secondary outcome measures

*Brief Symptom Inventory* (BSI; Dutch version by de Beurs (2011)) was used to assess comorbid psychological distress. This questionnaire consists of 53 items that measure general psychological distress. Patients score the items on a 5-point Likert scale. There are nine subscales for somatic complaints, cognitive problems, interpersonal sensitivity, depressive mood, anxiety, hostility, phobia, paranoia, and psychoticism. This questionnaire has good psychometrical features for older adults (de Beurs, 2011).

*Rosenberg Self-Esteem Scale* (RSES; Dutch version by Franck et al. (2008)) was used to assess comorbid psychological distress. The RSES consists of 10 items to measure self-esteem that are scored on a 4-point scale, from “totally agree” to “totally don’t agree.” A cutoff score < 15 means “low” self-esteem, a score between 15 and 25 means “average” self-esteem, and a score between 25 and 30 means “high” self-esteem. The questionnaire was highly reliable in mental healthcare patients aged 17 to 77 (Cronbach’s alpha = 0.89; Everaert et al., 2010).

### Measures filled out by the proxies

*Self-developed questionnaire* for knowledge, acceptance, and coping with autism spectrum disorder (ASD). This is for informants’ adjusted version of the questionnaire for patients. This questionnaire was also co-designed and adjusted after multiple conversations with a family experience expert.

The Dutch version of the *SRS-A, version for informants* (Constantino & Gruber, 2016), was used to assess coping with the diagnosis. The questionnaire consists of the same items as the self-report version of the SRS-A, but rephrased in a third-person point of view, like “he/she prefers to be alone rather than be with others.” The Dutch informant version is highly reliable (Cronbach’s alpha = 0.95, subscales between 0.81 and 0.89; test–retest reliability: r = 0.89, for subscales between 0.81 and 0.89).

#### Data analysis

First, to examine whether PE was related to improvements between pre-intervention (first session of the PE program), post-intervention (last session of the PE program), and follow-up (6 months after the PE program), a one-way repeated-measures analysis of variance (ANOVA) was used for the median scores of participants and informants. Examination of the variables at the different measurement points demonstrated that the data did not meet the parametric assumptions of normality, which led us to using the non-parametric alternative, the Friedman test.

Second, to examine whether the effects of the PE program as seen by the proxies differ from the effects observed by the participants, correlations of the difference scores between participants and proxies on the outcome measures on the different time points were used. We calculated the difference scores by subtracting the pre-intervention score from the post-intervention score, by subtracting the post-intervention score from the follow-up score, and by subtracting the pre-intervention score from the follow-up score. The relationship of the difference scores between the participants and proxies on the different outcomes was investigated using Spearman’s non-parametric correlation coefficient. According to Cohen (1992), the effect size is low if the value of r varies around 0.1, medium if r varies around 0.3, and large if r varies more than 0.5.

Finally, we used the results from the two qualitative open questions in our own questionnaire to investigate the participants’ and their proxies’ opinion about the PE program in more detail.

### Community involvement

Autistic older adults contributed to the development of our quantitative and qualitative measures in several feedback rounds. Furthermore, autistic older adults and clinicians working in the autism field contributed to our study by providing feedback on various decisions throughout the research project.

## Results

### Description of the sample

A total of 118 patients followed the PE program between May 2018 and May 2021 and were eligible to participate in the study. Seventy of the 118 patients and 44 proxies participated and filled in the questionnaires. Nine participants and three proxies were excluded because of lacking or incomplete consent to participate. This resulted in a total of 61 participants (mean age 66; range: 55–78) and 41 proxies (mostly a partner or child). Additional general information regarding the participants is depicted in Table 2. Specific data on socioeconomic status and educational attainment levels were not recorded.

**Table 2.** General characteristics of the participants.

Gender	N	Mean age	SD	Age range
Male	44	67.09	5.48	57–78
Female	17	63.24	4.90	55–75
Total	61	66.02	5.56	55–78

SD: standard deviation.

Attrition rate was especially high for the follow-up assessment. Only 33 of the 61 participants filled in the questionnaires at all three measurements and these data were used in the initial analyses. When considering only two measurements (pre-intervention and post-intervention), 48 participants filled in the questionnaires at both measurements. For the proxies, 23 of the 41 proxies filled in the questionnaires at all three measurements and were therefore considered in this study. When considering only two measurements (pre-intervention and post-intervention), 35 proxies filled in the questionnaires at both measurements.

For the qualitative analyses of the open questions in our own questionnaire, we used the answers of all 61 participants and 44 proxies.

### Results on the primary and secondary outcome measures

#### Results of the participants

Table 3 presents the median scores of the participants on the three different measurements and the results of the Friedman analysis.

**Table 3.** Overview of the median scores on the three measurements and the results of the Friedman test for the outcome measures for participants.

	N	Median M1	Median M2	Median M3	X^2^	p value	M1-M2 (p value)	M2-M3 (p value)	M1-M3 (p value)
Knowledge	32	1.67	2.11	2.43	33.168	0.000**	X^2^ = −0.891 (p = 0.001**)	X^2^ = −0.516 (p = 0.117)	X^2^ = −1.406 (p = 0.000**)
Acceptance	32	0.50	1.00	1.00	9.48	0.009**	X^2^ = −0.453 (p = 0.210)	X^2^ = −0.172 (p = 1.000)	X^2^ = −0.625 (p = 0.037**)
Coping	32	2.27	2.64	2.78	13.492	0.001**	X^2^ = −0.734 (p = 0.010**)	X^2^ = −0.078 (p = 1.000)	X^2^ = −0.812 (p = 0.003**)
SRS-A Total	33	80.50	80.50	74.50	5.225	0.073			
SRS-A Social Awareness	33	22.00	22.00	21.00	3.102	0.212			
SRS-A Social Communication	33	29.50	28.00	27.00	3.382	0.184			
SRS-A Social Motivation	33	16.00	16.50	16.00	0.617	0.735			
SRS-A Repetitiveness and Rigidity	33	12.00	12.00	12.50	0.624	0.732			
BSI Total	31	1.15	1.18	1.08	5.175	0.075			
BSI Somatic Complaints	32	0.57	0.64	0.71	0.120	0.942			
BSI Cognitive Complaints	32	1.67	1.75	1.33	3.185	0.203			
BSI Interpersonal symptoms	32	1.38	1.25	1.00	3.321	0.190			
BSI Depression	32	1.33	1.18	1.00	3.105	0.212			
BSI Anxiety	31	1.33	1.00	1.17	1.273	0.529			
BSI Hostility	32	0.80	0.60	0.60	8.608	0.014**	X^2^ = 0.438 (p = 0.240)	X^2^ = 0.203 (p = 1.000)	X^2^ = 0.641 (p = 0.031**)
BSI Fear	31	1.00	0.80	1.000	4.551	0.103			
BSI Paranoia	32	1.00	0.80	0.900	3.135	0.209			
BSI Psychoticism	31	1.20	1.00	1.000	2.775	0.250			
RSES	32	15.00	13.00	10.50	9.868	0.007**	X^2^ = 0.609 (p = 0.044**)	X^2^ = 0.094 (p = 1.000)	X^2^ = 0.703 (p = 0.015**)

M1: pre-intervention; M2: post-intervention; M3: follow-up; SRS-A: Social Responsiveness Scale–Adults; BSI: Brief Symptom Inventory; RSES: Rosenberg Self-Esteem Scale.

** *p* < 0.05.

##### Knowledge

A statistically significant increase was found in the scores of the subscale Knowledge of our questionnaire across the three time points, with a medium to large effect size (χ^2^ = −1.406, p = 0.000; r = 0.45–0.70).

##### Acceptance

We observed a significant increase in the acceptance scores of our questionnaire across the three time points (χ^2^ = −0.625, p = 0.037). Post hoc tests (with Bonferroni correction for multiple tests) indicated that there was a significant increase from pre-intervention to follow-up, with a medium effect size (r = 0.31).

##### Coping

The results of the Friedman test indicated that there was a statistically significant increase in the scores of the participants on the subscale Coping of our questionnaire across the three time points, with a medium effect size (χ^2^ = −.812, p = 0.003; r = 0.37–0.41).

##### Psychological distress

There was a statistically significant decrease in the Hostility scores of the BSI across the three time points, with a medium effect size (χ^2^ = 0.641, p = 0.031; r = 0.32). This implies that the participants of the PE program experienced a significant improvement in the hostility and anger symptoms pre-intervention versus follow-up. There was no significant difference in the overall psychological distress of participants (χ^2^ = 5.175, p = 0.075), nor in other symptoms of distress. Second, and unexpectedly, we found a statistically significant decrease in the self-esteem scores on the RSES across the three time points, with a medium effect size (χ^2^ = 0.703, p = 0.015; r = 0.30–0.35).

#### Results of the proxies

Table 4 presents the median scores of the informants on the three different measurements for the different outcome measures and the results of the Friedman analysis.

**Table 4.** Median scores and results of the Wilcoxon signed rank test for the outcome measures for participants on M1 and M2.

Var	N	Z	p value	Median M1	Median M2
Knowledge	47	−4.440	<0.001**	1.67	2.11
Acceptance	47	−0.757	0.449	0.50	1.00
Coping	46	−3.898	<0.001**	2.27	2.64
SRS-A Total	48	−2.393	0.017**	80.50	80.50
SRS-A Social Awareness	48	−2.250	0.024**	22.00	22.00
SRS-A Social Communication	48	−2.166	0.030**	29.5	28.00
SRS-A Social Motivation	48	−1.573	0.116	16.00	16.50
SRS-A Repetitiveness and Rigidity	48	−0.54	0.589	12.00	12.00
BSI Total	47	−2.346	0.019**	1.15	1.18
BSI Somatic Complaints	48	−0.892	0.372	0.57	0.64
BSI Cognitive Complaints	48	−1.931	0.054	1.67	1.75
BSI Interpersonal symptoms	48	−1.516	0.130	1.38	1.25
BSI Depression	48	−2.514	0.012**	1.33	1.18
BSI Anxiety	47	−0.386	0.699	1.33	1.00
BSI Hostility	48	−2.593	0.010**	0.80	0.60
BSI Fear	47	−2.002	0.045**	1.00	0.80
BSI Paranoia	47	−1.453	0.146	1.00	0.80
BSI Psychoticism	47	−1.329	0.184	1.20	1.00
RSES	47	−2.342	0.019**	15.00	13.00

M1: pre-intervention; M2: post-intervention; SRS-A: Social Responsiveness Scale–Adults; BSI: Brief Symptom Inventory; RSES: Rosenberg Self-Esteem Scale.

** *p* < 0.05.

##### Knowledge

We observed a statistically significant increase for the scores of the subscale Knowledge of our questionnaire for proxies across the three time points, with a medium effect size (χ^2^ = −0.937, p = 0.003; r = 0.47).

##### Acceptance

We observed a significant increase in the acceptance scores of our questionnaire for proxies across the three time points with a small effect size (χ^2^ = −0.208, p = 1.000; r = 0.15). No significant differences were found between the time points on the post hoc tests.

##### Coping

There was a statistically significant increase in the scores of the proxies on the subscale Coping of our questionnaire across the three time points, with a large effect size (χ^2^ = −1.174, p = 0.000**; r = 0.52–0.59).

Furthermore, we observed a significant increase in the SRS-A score for the subscale SM for proxies between the three different time points, with a medium to large effect size (χ^2^ = 0.848, p = 0.012; r = 0.42–0.52).

### Re-analyses for pre-intervention to post-intervention effects

Due to our relatively small group size for participants and proxies at follow-up, we repeated the analyses for only two time points (pre-intervention and post-intervention), resulting in a larger group size. We used the non-parametric equivalent of the paired-samples t test, the Wilcoxon signed rank test (see Tables 4 and 5). For participants, we found significant increases with a large effect size on knowledge about the diagnosis (Z = −4.44, p < 0.001; r = 0.68) and coping with the diagnosis on our own questionnaire (Z = −3.90, p < 0.001; r = 0.57). We found significant increases with medium effect sizes on the SRS-A total score (Z = −2.39, p = 0.017; r = 0.35), SRS-A SA (Z = −2.25, p = 0.024; r = 0.32), SRS-A SC (Z = −2.17, p = 0.030; r = 0.32), and decreases with medium effect sizes on psychological distress (BSI total score: Z = −2.35, p = 0.018; r = 0.32; depression: Z = −2.51, p = 0.012, r = 0.37; hostility: Z = −2.59, p = 0.010, r = 0.38; and fear: Z = −2.00, p = 0.045, r = 0.29) and on self-esteem (RSES: Z = 2.34, p = 0.019, r = 0.34).

**Table 5.** Results of the Wilcoxon signed rank test for outcome measures for proxies on M1 and M2.

Var	N	Z	p value	Median M1	Median M2
Knowledge	35	−4.369	<0.001**	1.82	2.27
Acceptance	35	−2.443	0.015**	0.75	1.00
Coping	33	−4.369	<0.001**	1.82	2.27
SRS-A Total	35	−3.415	<0.001**	85.00	74.00
SRS-A Social Awareness	35	−1.640	0.101	23.00	22.50
SRS-A Social Communication	35	−3.112	0.002**	29.00	26.00
SRS-A Social Motivation	35	−3.280	0.001**	16.00	14.00
SRS-A Repetitiveness and Rigidity	35	−2.405	0.016**	14.00	11.00

M1: pre-intervention; M2: post-intervention; SRS-A: Social Responsiveness Scale-Adults.

** *p* < 0.05.

For the proxies we found significant increases with medium to large effect sizes on knowledge about the diagnosis (Z = −4.37, p < 0.001; r = 0.74), acceptance of the diagnosis (Z = −2.44, p = 0.015, r = 0.41), coping with the diagnosis (Z = −4.37, p < 0.001, r = 0.74), the SRS-A total score (Z = −3.42, p < 0.001, r = 0.58), SRS-A SC (Z = −3.11, p = 0.002, r = 0.53), SRS-A SM (Z = −3.11, p = 0.001, r = 0.53), and SRS-A RR (Z = −2.41, p = 0.016, r = 0.41).

### Difference between participants and proxies

Online Table A in the supplemental appendix shows the results for the Spearman non-parametric correlation coefficient between the difference scores of the participants and proxies on the different outcome measures (our questionnaire and SRS-A) pre-intervention (M1) versus post-intervention (M2) versus 6-month follow-up (M3).

There was a strong, positive correlation between the difference scores of the participants and proxies on the subscale Knowledge of our questionnaire pre-intervention and post-intervention, with participants and proxies both observing an increase in knowledge post-intervention in comparison with pre-intervention (rho = 1.00, p < 0.001). Furthermore, there was a medium, positive correlation between the difference scores on Knowledge of participants and proxies post-intervention and at 6-month follow-up (rho = 0.433, p = 0.039). Furthermore, we found medium, positive correlations between the difference scores of the participants and proxies on the subscale SA of the SRS-A pre-intervention and post-intervention (rho = 0.379, p = 0.032), and pre-intervention versus at 6-month follow-up (rho = 0.470, p = 0.027).

### Qualitative analyses for participants and proxies

Post-intervention, almost all participants reported on the open questions that the PE program had improved their knowledge, insight, and understanding of autism and that their coping with autistic traits had improved. Typical quotes were as follows: “I have gained more insight and have become more open in communication,” “I think I have learned a lot,” and “I am not insane, my brain just works differently.”

The PE program was also helpful for participants in recognizing how being autistic was impacting themselves and their environment. At follow-up, it was observed that most participants experienced an improvement in acceptance of the diagnosis and in insight and understanding of the diagnosis. They also reported recognition of how being autistic was impacting themselves and their environment. An example is, “She had more knowledge about her limitations because of her autism in daily life, and she can cope better.” Only one participant at post-intervention and one participant at follow-up reported a lack of insight and understanding. Overall, we can conclude that the vast majority of participants gave positive feedback on the PE program.

Post-intervention, most participants reported on the open questions that the PE program had helped them in recognizing how being autistic was impacting them. Typical quotes are as follows: “I accept myself more as being different and I have realized that I have more to offer than I first thought,” and “I have gained a better understanding of my autism and the consequences in communicating with others; communication has improved and there are less conflicts in my relationship with my wife.” Also, recognition of group members was reported as helpful. At follow-up, most proxies observed an improvement in the knowledge of autism, and insight and understanding by the participants. Only one proxy reported a lack of insight and understanding at follow-up for the participant. Post-intervention, three proxies reported they observed no effect of the PE program for the participant, and at follow-up this was reported by only one proxy. Overall, we can conclude that most of the proxies gave positive feedback on the PE program.

## Discussion

In this study, the effects of the PE program “Me and Autism 60+” for older adults were explored. First, we found that knowledge of autism among the participants improved significantly. Proxies also observed these improvements in knowledge in the participants, with a strong and significant correlation between participants and proxies.

Second, participants experienced an improvement in acceptance of the diagnosis. However, at 6-month follow-up, a further improvement in acceptance of the diagnosis was not observed. Proxies also observed a small improvement in acceptance of the diagnosis by the participant, but this result was not significant.

Third, there was an improvement in coping as measured with our self-developed questionnaire. Also, according to the proxies, the participants’ coping with autistic traits did improve as measured with our questionnaire. Proxies also observed a significant improvement in social motivation of the participants on the SRS-A, that is, their willingness to participate in social interactions. We found only one significant medium, positive relation between participants and proxies for coping with the diagnosis on the SRS-A, that is, for SA, the ability to recognize and interpret social cues.

Fourth, no improvement was observed in psychological distress of the participants. Participants only experienced an improvement in their symptoms of anger and hostility. Unexpectedly, we found a decrease in self-esteem.

Finally, the qualitative questions showed that most participants and proxies gave positive feedback on the PE program. Increase in insight and understanding, coping with and acceptance of autism, and recognizing how autism works for them personally were the most frequently reported findings by participants and proxies. Contact with other autistic older adults was experienced as helpful.

Our hypotheses on the effects on coping and acceptance were partly confirmed, and there were no positive effects on comorbid symptoms. Possibly, our expectations might have been too high, as the PE program followed shortly after the autism diagnosis. The program is a first step in the treatment of autistic older adults and therefore focuses on information about the diagnosis and recognizing how autism is impacting the older adult’s life and his or her interaction with others, rather than focusing on treatment of autism-related symptoms, problem-solving, and coping with comorbid symptoms. Moreover, possibly, the PE program helps the older adult with a (very) late diagnosis of autism to review his or her life in the context of this diagnosis (Stagg & Belcher, 2019).

A possible explanation for the observed improvement in coping according to the proxies, which was not experienced by the participants themselves, that is, in social motivation of the participant, might be the more limited self-reflective capabilities of autistic older adults in comparison with informants without autism.

The decrease in self-esteem, despite the improved acceptance of the diagnosis, was contrary to our expectations. It could be explained by the fact that, because participants gained knowledge and insight, they might have realized that the consequences of the diagnosis in daily life were still present—and had been present during the long life they had lived (e.g. relationship problems, feelings of being different). This may have led to a life review and feelings of guilt and grief, which in turn affected self-esteem. Furthermore, the social image of autism is mainly negative, even stigmatizing, and focuses on problems and limitations (Turnock et al., 2022). Especially, older adults have adopted these negative qualifications, as autism has long been mainly associated with intellectual disability and older adults have been confronted with this negative image for decades (Robison, 2019).

### Methodological considerations and future research

This study has several limitations. First, our total group sample was relatively large for an exploratory study, but attrition rate was high for the follow-up assessment. Possibly, those participants who did fill out the follow-up measures are a selective group, who were more able or willing to reflect, or were more inclined to participate as they were more positive.

Second, after completion of the PE program, some participants followed a treatment program, other participants did not. There may be a difference in improvement on the different outcome measures at follow-up between the participants that received a further treatment and those who did not.

Third, our own self-developed questionnaire for knowledge, coping, and acceptance has not been validated yet. It was developed though in co-creation with older autistic experience experts and a family experience expert, in order to maximize comprehension by older autistic adults.

Fourth, unfortunately, the Covid-19 pandemic had its course during this study. The treatment sessions were canceled for months during the lockdown in the Netherlands, and afterward, we had to reduce the number of participants per group from eight to six participants, in order to take social distancing into account. This limited the opportunities for participants to have contact with other group members, with possible negative effects on the recognition of group members. Furthermore, because of social distancing measures, it was no longer possible for participants to invite a proxy to the eighth session of the program. This has affected half of the participants and proxies and might, in turn, have affected the results. Moreover, negative effects of the Covid-19 pandemic on quality of life have been found in autistic adults (Scheeren et al., 2022, 2023), so also for our participants, this may have affected the outcomes on our assessments, most likely on psychological distress.

Fifth, it should also be taken into account that it is possible that the self-report questionnaire BSI is not sufficiently sensitive to measure subtle changes in comorbid complaint levels of autistic participants. The total BSI score of the participants in this study (mean score = 1.28, a score between the minimum of 0 and a maximum of 4) was lower than expected, given the findings of Lever and Geurts (2016) that approximately 69% of the autistic adult population suffer from comorbid psychiatric disorders. Thus, comorbid psychiatric disorders may have acted as possible confounders. A recommendation for future research would be to consider using a different outcome measure that is known to be able to measure subtle changes in comorbid symptoms in autistic people. Also, the SRS-A is designed as a screening instrument for autism. It also measures changes in autistic behavior but may not be sensitive for subtle changes in autistic behavior. The AQ-short version (Hoekstra et al., 2011), superior to the full AQ in older adults (Agelink van Rentergem et al., 2019), although also mainly a screening instrument, could be a possible alternative. Additional outcome measures for future studies could be quality of life (Mental Health Quality of Life-7; van Krugten et al., 2022) or recovery (Individual Recovery Outcomes Counter; Monger et al., 2013).

Furthermore, an important limitation for this study is the lack of a control group. As it may be considered unethical not to provide PE shortly after an autism diagnosis, in future research, we recommend using some control group, for instance, a waiting list condition.

Finally, and perhaps the most important reflection on the PE program “Me and Autism 60+” that was studied is that it was developed a decade ago, and at the time, the assumption was that (older) adults needed to be educated about what autism is. Over the past few years, this assumption has changed. The idea at the time, in these traditional programs, was that after receiving an autism diagnosis, people were to be provided information on classical autism theories. Not only have the theories about autism evolved (Happé & Frith, 2020; Pellicano & den Houting, 2022). It might be much more important that participants are helped at recognizing how being autistic is impacting themselves and their environment. It also addresses the question what the primary outcome of a PE intervention actually should be. This might very well be helping older adults to construct a (new) narrative of themselves, and the life they have lived, in the light of the recent autism diagnosis. Furthermore, it might be helpful to also focus on age-related topics in this program (Groenendijk et al., 2022).

This study also has some strengths. It is the first “larger” explorative study into PE for autistic older adults. Also, treatment adherence was secured, so that the same treatment content was provided in the PE groups.

## Conclusion

This exploratory study of a PE program for autistic older adults did yield some promising indications, quantitatively and qualitatively, for its effectiveness. Further research into the effects of this PE program is necessary. Hence, further adaptation of the program, based on the feedback participants and proxies gave, is a worthwhile pursuit. In redesigning this PE intervention, a more helpful primary outcome might very well be helping older adults with a late autism diagnosis to construct a (new) narrative of themselves, and the life they have lived. But for now, we advise, in accordance with the Multidisciplinary Guideline and with the positive feedback of the participants and informants, to use this adapted PE program for older adults after a late diagnosis of ASD, just as PE is provided as standard care to younger cohorts after receiving an autism diagnosis in the Netherlands: first things first.

## Supplemental Material

sj-docx-1-aut-10.1177_13623613231219745 – Supplemental material for First things first: An exploration of the effects of psychoeducation for older autistic adults Supplemental material, sj-docx-1-aut-10.1177_13623613231219745 for First things first: An exploration of the effects of psychoeducation for older autistic adults by Maartje Lenders, Machteld A Ouwens, Rosalien M H J Wilting and Arjan C Videler in Autism
