# Supplementary material for: First things first: An exploration of the effects of psychoeducation for older autistic adults
Source: Autism. 2024 Jan 10;28(8):2028–39. doi: 10.1177/13623613231219745 (PMC11301958; doi:10.1177/13623613231219745)
Supplement: sj-docx-1-aut-10.1177_13623613231219745 – Supplemental material for First things first: An exploration of the effects of psychoeducation for older autistic adults [file sj-docx-1-aut-10.1177_13623613231219745.docx]

Online Table A

*An overview of the results of the correlations between participants and informants*

| **Difference score participant-informant for variable** | **N** | **Correlation**  **Spearman’s rho** | **Significance p-value** |
| --- | --- | --- | --- |
| Our Knowledge M1-M2 | 35 | 1.000^**^ | .000** |
| Our Knowledge M2-M3 | 23 | .433^**^ | .039^**^ |
| Our Knowledge M1-M3 | 22 | .039 | .862 |
| Our Coping M1-M2 | 29 | .110 | .569 |
| Our Coping M2-M3 | 23 | -.127 | .565 |
| Our Coping M1-M3 | 21 | .005 | .984 |
| Our Acceptance M1-M2 | 31 | -.028 | .883 |
| Our Acceptance M2-M3 | 23 | -.171 | .434 |
| Our Acceptance M1-M3 | 22 | -.002 | .992 |
| SRS-A Total M1-M2 | 32 | .258 | .154 |
| SRS-A Total M2-M3 | 22 | .368 | .092 |
| SRS-A Total M1-M3 | 22 | .092 | .684 |
| SRS-A Social Awareness M1-M2 | 32 | .379** | .032** |
| SRS-A Social Awareness M2-M3 | 22 | .192 | .393 |
| SRS-A Social Awareness M1-M3 | 22 | .470** | .027^**^ |
| SRS-A Social Communication M1-M2 | 32 | .113 | .540 |
| SRS-A Social Communication M2-M3 | 22 | .318 | .149 |
| SRS-A Social Communication M1-M3 | 22 | .028 | .900 |
| SRS-A Social Motivation M1-M2 | 32 | .264 | .144 |
| SRS-A Social Motivation M2-M3 | 22 | .104 | .646 |
| SRS-A Social Motivation M1-M3 | 22 | -.050 | .825 |
| SRS-A Repetitiveness and Rigidity M1-M2 | 32 | -.205 | .260 |
| SRS-A Repetitiveness and Rigidity M2-M3 | 22 | -.024 | .916 |
| SRS-A Repetitiveness and Rigidity M1-M3 | 22 | -.095 | .675 |

*M1 = pre-intervention; M2 = post-intervention; M3 = follow up; ** p<.05*
